# An Atypical Finding in a Patient With Acute Left Flank Pain

**DOI:** 10.7759/cureus.112267

**Published:** 2026-07-08

**Authors:** Achraf Rhallab, Sandy Van Nieuwenhove

**Affiliations:** 1 Radiology, Cliniques Universitaires Saint-Luc, Brussels, BEL

**Keywords:** acute flank pain, adrenal hemorrhage, adrenal infarction, computed tomography, diffusion restriction, hemorrhagic transformation, imaging pitfall, magnetic resonance imaging

## Abstract

Hemorrhagic adrenal infarction is a rare and potentially under-recognized cause of acute flank pain. We report the case of a 29-year-old woman presenting with acute left flank pain of two days’ duration. Initial contrast-enhanced abdominal CT, including unenhanced and portal venous phases, was interpreted as normal. Retrospective review demonstrated subtle enlargement of the left adrenal gland with mildly reduced enhancement compared with the contralateral gland.

Following clinical deterioration with fever, a repeat CT performed five days later showed marked enlargement of the left adrenal gland with absent enhancement and surrounding periadrenal fat stranding, consistent with hemorrhagic adrenal infarction. MRI confirmed heterogeneous signal intensity, diffusion restriction, and lack of post-contrast enhancement, supporting the diagnosis.

Laboratory evaluation revealed severe primary hypothyroidism consistent with autoimmune thyroiditis and heterozygous hemoglobin S (sickle cell trait). Endocrine assessment showed preserved adrenal function without evidence of adrenal insufficiency. Initial thrombophilia testing demonstrated abnormalities in coagulation factors interpreted in the context of an acute inflammatory state, and was considered non-contributory on hematology review.

The patient was managed conservatively following multidisciplinary discussion, without anticoagulation, and showed favorable clinical and radiological evolution.

This case highlights an important imaging pitfall: early unilateral adrenal infarction may present with subtle CT findings and can be missed on initial interpretation. Careful adrenal evaluation and short-interval follow-up imaging are essential in patients with persistent or worsening acute flank pain despite an initially negative CT.

## Introduction

Hemorrhagic adrenal infarction is a rare and potentially under-recognized cause of acute flank or abdominal pain [1,2]. The adrenal glands are highly vascular endocrine organs supplied by multiple arterial branches but drained by a single central vein, creating a relative venous bottleneck that predisposes them to congestion and ischemic-hemorrhagic events under systemic stress.

Although classically described in critically ill patients, adrenal hemorrhage and infarction may also occur in otherwise healthy individuals, often presenting with nonspecific clinical symptoms [1].

Early computed tomography (CT) findings may be subtle and can be misinterpreted as normal, particularly in unilateral cases [3,4]. The differential diagnosis of acute flank pain is broad and includes renal colic, pyelonephritis, splenic infarction, adrenal neoplasm or hemorrhagic mass, and retroperitoneal inflammatory processes, which further complicates timely diagnosis.

Given the nonspecific clinical presentation, adrenal infarction may be overlooked if the adrenal glands are not carefully evaluated. Early recognition is essential to avoid misdiagnosis, unnecessary investigations, and delayed follow-up, particularly to exclude an underlying adrenal lesion and to monitor for potential adrenal insufficiency in bilateral disease. We report a case of unilateral hemorrhagic adrenal infarction initially missed on CT, with subsequent hemorrhagic evolution demonstrated on follow-up imaging.

## Case presentation

A 29-year-old woman presented to the emergency department with acute left flank pain of two days’ duration (pain intensity 9/10). She had no relevant past medical history and denied anticoagulant use, oral contraceptives, or hormonal therapy. Pregnancy was excluded by negative serum β-human chorionic gonadotropin (hCG).

On admission, vital signs were stable. During hospitalization, she developed a fever up to 38.2°C. She denied recent trauma, dehydration, travel, or preceding systemic infectious symptoms. Physical examination revealed localized tenderness over the left flank.

Laboratory findings are summarized in Table 1. Initial laboratory testing showed elevated inflammatory markers and lactate dehydrogenase. Urinalysis demonstrated leukocyturia and microscopic hematuria, and urine culture grew *Citrobacter koseri*, for which empirical antibiotic therapy was initiated for presumed urinary tract infection. Thyroid function testing revealed severe primary hypothyroidism with positive anti-thyroid peroxidase and anti-thyroglobulin antibodies, consistent with autoimmune thyroiditis. Hemoglobin electrophoresis demonstrated heterozygous hemoglobin S (39.8%).

**Table 1:** Laboratory findings on admission. ACTH = adrenocorticotropic hormone; Anti-TPO = anti-thyroid peroxidase antibodies; CRP = C-reactive protein; Free T4 = free thyroxine; HbS = hemoglobin S; TSH = thyroid-stimulating hormone; WBC = white blood cells

Parameter	Result	Units	Reference Range
Hemoglobin	12.1	g/dL	12.0-16.0
White blood cells	8.16	×10³/µL	4.0-10.0
Platelets	288	×10³/µL	150-350
C-reactive protein (CRP)	118	mg/L	<5
Fibrinogen	684	mg/dL	150-450
D-dimer	1,566	ng/mL	<500
Sodium	138	mmol/L	135-145
Potassium	3.58	mmol/L	3.5-5.0
Cortisol (morning)	300.4	nmol/L	171-536
ACTH	55.7	pg/mL	5-49
Thyroid-stimulating hormone (TSH)	122	mIU/L	0.30-4.20
Free T4	7.9	pmol/L	12-22
Anti-TPO antibodies	>600	U/mL	<34
Anti-thyroglobulin antibodies	612	U/mL	<115
Hemoglobin S (HbS fraction)	39.8	%	Absent

Serum cortisol and electrolytes were within normal limits. Adrenocorticotropic hormone (ACTH) was mildly elevated (55.7 pg/mL), interpreted as a transient stress-related response during acute illness, with no biochemical evidence of adrenal insufficiency.

A thrombophilia workup was reviewed in hematology consultation and was considered unremarkable; however, complete primary laboratory documentation was not available for independent verification at the time of manuscript preparation.

A contrast-enhanced abdominal CT was performed, including unenhanced and portal venous phases. Image analysis included region-of-interest measurements within the adrenal glands, avoiding central vessels and adjacent fat, on thin-section reconstructions.

**Figure 1:**
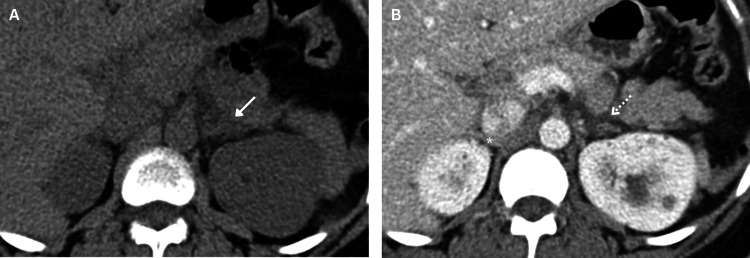
Initial CT (unenhanced and contrast-enhanced phases) demonstrating left adrenal hemorrhagic infarction. (A) Unenhanced CT shows subtle enlargement of the left adrenal gland (maximum limb thickness: 9 mm) (arrow) with increased attenuation on the left (~50 HU) compared with the right adrenal gland (maximum limb thickness: 3 mm; ~35 HU) (not shown). (B) Portal venous phase shows mild hypoenhancement of the left adrenal gland (dotted arrow) compared with the contralateral side (asterisk), with heterogeneous attenuation in the left adrenal gland (~22-110 HU), including early low-attenuation areas (~22 HU) and focal relatively hyperenhancing components (up to ~110 HU), while the contralateral adrenal demonstrates homogeneous enhancement (~100 HU).

The initial radiological interpretation was reported as normal. Retrospective review demonstrated subtle enlargement of the left adrenal gland with mildly reduced enhancement compared with the contralateral gland (Figure 1).

**Figure 2:**
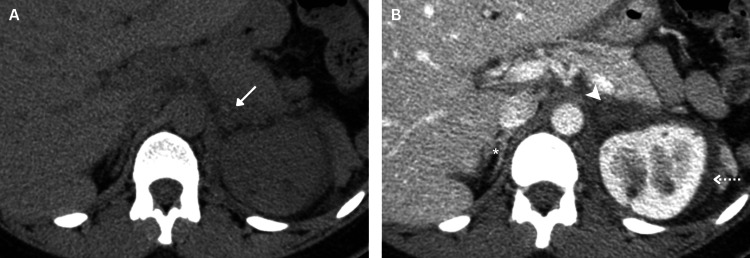
Follow-up contrast-enhanced CT performed five days later. (A) Unenhanced CT shows marked enlargement of the left adrenal gland (maximum limb thickness: 13 mm vs. 3 mm contralaterally) (arrow). (B) Portal venous phase demonstrates a complete absence of enhancement of the left adrenal gland (arrowhead) with surrounding periadrenal fat stranding (dotted arrow). The gland shows heterogeneous attenuation with low-density areas (~17 HU) and residual faint enhancing components (~75 HU) (not shown in this slice), compared with the contralateral adrenal gland (~100 HU) (asterisk).

Five days later, due to worsening pain and persistent fever, a follow-up contrast-enhanced CT was performed, demonstrating marked enlargement of the left adrenal gland with absence of enhancement and surrounding periadrenal and perirenal fat stranding, consistent with hemorrhagic adrenal infarction (Figure 2).

**Figure 3:**
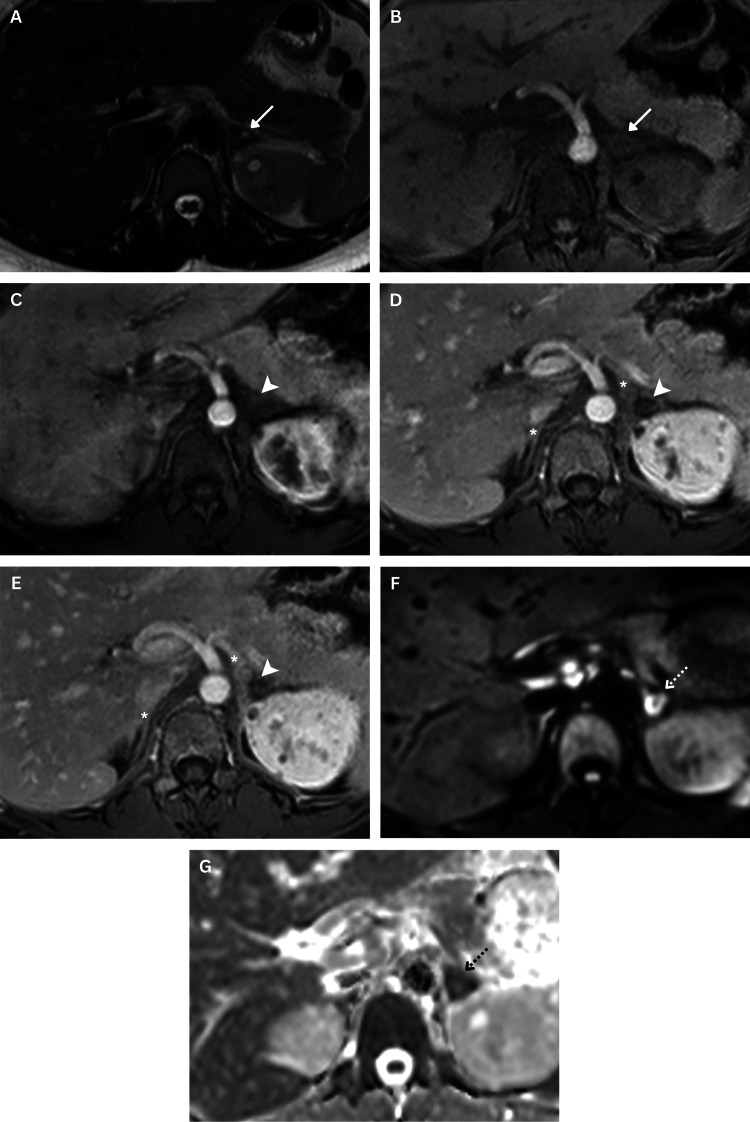
Follow-up magnetic resonance imaging. (A) Axial T2-weighted image and (B) axial pre-contrast T1-weighted image demonstrate heterogeneous signal intensity within the left adrenal gland (arrows). (C-E) Axial post-contrast T1-weighted mDixon images show a focal area of non-enhancement within the left adrenal gland (arrowheads), with preserved enhancement of the remaining left adrenal tissue and the contralateral adrenal gland (asterisks). (F) Diffusion-weighted imaging demonstrates high signal intensity with corresponding low signal on the apparent diffusion coefficient map (G), consistent with diffusion restriction and supporting the diagnosis of adrenal infarction with hemorrhagic transformation.

MRI performed two months later demonstrated persistent enlargement of the left adrenal gland (maximum limb thickness: 13 mm) with heterogeneous signal intensity on both T1- and T2-weighted sequences, consistent with hemorrhagic transformation. Diffusion-weighted imaging showed high signal with corresponding low apparent diffusion coefficient values. Post-contrast images demonstrated the absence of enhancement in the affected region (Figure 3). Follow-up MRI showed near-complete regression.

The patient was managed conservatively following multidisciplinary discussion involving endocrinology and hematology teams. Anticoagulation was not initiated due to the hemorrhagic nature of the lesion. Levothyroxine replacement therapy was started, with progressive clinical improvement and a favorable radiological outcome.

## Discussion

Pathophysiology

Hemorrhagic adrenal infarction results from the distinctive vascular architecture of the adrenal gland. Multiple arterial branches form a dense subcapsular plexus that drains through a limited number of venous channels into a single central vein. This configuration creates a functional “vascular dam,” predisposing the gland to venous congestion, thrombosis, ischemia, and secondary hemorrhagic transformation in the context of physiological stress or hypercoagulable states [1-3].

Hemoglobin electrophoresis in this patient demonstrated heterozygous hemoglobin S (sickle cell trait). Although generally considered a benign carrier state, it has been associated with microvascular complications under conditions such as hypoxia, dehydration, or systemic inflammation [5]. Given the adrenal gland’s terminal vascular supply, sickle cell trait may represent a susceptibility factor in the setting of acute systemic stress; however, a causal relationship cannot be inferred from a single case.

Severe hypothyroidism has also been associated with alterations in haemostatic balance, including reduced fibrinolysis and a tendency toward a prothrombotic state. Nevertheless, direct evidence linking hypothyroidism to adrenal infarction remains limited, and in this context, autoimmune thyroiditis should be regarded as a potential associated condition rather than a proven etiological factor.

Epidemiology and etiology

In a large 25-year Mayo Clinic series, adrenal hemorrhage was most frequently observed in hospitalized or critically ill patients and was typically associated with systemic stress [1]. More recent data suggest that adrenal hemorrhage is predominantly unilateral, whereas bilateral involvement is less common [2]. Reported etiologies include postoperative states, trauma, anticoagulation, coagulopathy, adrenal neoplasms, and sepsis [2,3]. Spontaneous and atraumatic cases have also been described, often in association with inflammatory or prothrombotic conditions [6,7].

Imaging findings

Contrast-enhanced CT is the imaging modality of choice in the acute setting. Typical findings include adrenal enlargement, heterogeneous attenuation, preserved gland morphology in early stages, and periadrenal fat stranding [4]. Subtle enlargement with relatively decreased enhancement may represent an early imaging sign preceding overt hemorrhagic transformation [3]. Awareness of these early and often subtle findings is crucial to avoid misinterpretation and delayed diagnosis [8].

In pediatric populations, adrenal hemorrhage should be distinguished from neuroblastoma, which may present as a suprarenal mass with overlapping imaging features [9].

Endocrine implications

Adrenal insufficiency is primarily associated with bilateral adrenal involvement. Across published series, endocrine dysfunction is uncommon in unilateral disease, where the contralateral gland usually maintains adequate hormonal function [1,2,10]. In the present case, cortisol levels and electrolytes remained within normal limits, and no clinical or biochemical evidence of adrenal insufficiency was identified, consistent with preserved contralateral adrenal function.

Management and clinical implications

Unilateral hemorrhagic adrenal infarction is most often managed conservatively [2]. Radiological follow-up typically demonstrates gradual resolution over time, occasionally with residual atrophic or cystic changes [2,4]. This case highlights that early unilateral adrenal infarction may present with only subtle CT abnormalities and can be initially overlooked. Careful systematic evaluation of the adrenal glands and short-interval follow-up imaging are therefore essential in patients with persistent acute flank pain, particularly in the presence of systemic inflammation or potential susceptibility factors.

## Conclusions

Unilateral hemorrhagic adrenal infarction may present with subtle findings on initial CT and can be misinterpreted as normal. Recognition of asymmetric adrenal enlargement and reduced enhancement, together with short-interval follow-up imaging when clinical suspicion persists, is essential for accurate diagnosis. Microvascular susceptibility factors, such as sickle cell trait, and associated systemic conditions, including severe hypothyroidism, may represent potential contributing factors; however, their causal role remains unproven and should be interpreted with caution.

Follow-up imaging is important to document lesion regression and to exclude an underlying adrenal neoplasm. Careful assessment of the adrenal glands should be considered in patients presenting with persistent acute flank pain, even when initial imaging appears unremarkable.
